# Assessing the role of plan complexity and target geometry through multi‐institutional gel based end‐to‐end QA in multi‐focal single isocenter stereotactic radiosurgery

**DOI:** 10.1002/acm2.70758

**Published:** 2026-09-24

**Authors:** Despoina Stasinou, Kalliopi Platoni, Vasiliki Margaroni, Kyveli Zourari, Emmanouil Zoros, Niko Papanikolaou, Evangelos Pappas

**Affiliations:** ^1^ Department of Biomedical Sciences, Radiology and Radiotherapy Sector University of West Attica Athens Attica Greece; ^2^ Department of Applied Medical Physics Medical School, Attikon University Hospital National and Kapodistrian University of Athens Athens Attica Greece; ^3^ Medical Physics Laboratory School of Medicine National and Kapodistrian University of Athens Athens Attica Greece; ^4^ Department of Applied Medical Physics University General Hospital of West Attica “H AGIA VARBARA” Athens Attica Greece; ^5^ Department of Radiation Oncology The University of Texas Health Science Center at San Antonio San Antonio Texas USA; ^6^ School of Biomedical Engineering and Imaging Sciences King's College London, London England UK

**Keywords:** anthropomorphic phantom, complexity metrics, end‐to‐end test, gel dosimetry, multi‐institutional study, multiple brain metastases, single isocenter SRS

## Abstract

**Background:**

Single‐isocenter stereotactic radiosurgery enables efficient treatment of multiple brain metastases (SI‐MBM SRS), but demands high geometric accuracy. Plan complexity metrics are increasingly used as indicators for quality assurance (QA) performance, however their applicability to SRS remains uncertain, particularly in the context of multi‐institutional variability.

**Purpose:**

This study evaluated the relationship between plan complexity, target geometry, and end‐to‐end dosimetric QA outcomes for SI‐MBM SRS across multiple institutions.

**Methods:**

Forty‐two SI‐MBM SRS plans from different centers and platforms were delivered to polymer gel phantoms, providing high‐resolution 3D dose measurements. Gamma passing rates (GPRs) were calculated under 3%/2 mm, 5%/2 mm and 5%/1 mm criteria and were correlated with eleven established complexity metrics calculated per plan. Geometric factors, including target equivalent diameter and distance‐to‐isocenter, were analyzed. Receiver‐operating‐characteristic (ROC) analysis was performed to identify optimal thresholds for predicting QA pass/fail (≥90% GPR).

**Results:**

No statistically significant differences between the different linacs and treatment planning systems were found, nor strong or consistent correlations between complexity metrics and GPRs. In contrast, geometric parameters were more influential: off‐axis distance and target size significantly affected QA performance, with the largest differences observed for far‐off‐axis lesions. ROC analysis identified optimal thresholds of 34.9 mm for distance‐to‐isocenter and 4.8 mm for equivalent diameter in predicting QA outcomes (AUC∼0.60‐0.65), although the predictive performance remained modest.

**Conclusions:**

In this multi‐institutional, gel‐based end‐to‐end study of SI‐MBM SRS, target geometry was a stronger QA performance predictor than complexity metrics. These findings emphasize the importance of geometry‐aware QA strategies and the need for further standardized, multi‐institutional evaluations to clarify the interplay between complexity, geometry, and machine performance in SRS.

## 1 INTRODUCTION

Stereotactic radiosurgery (SRS) is a well‐established treatment modality for intracranial malignancies, delivering high‐dose radiation with high conformity and precision.^1^, ^2^ While traditionally used for solitary lesions, advancements in treatment planning, imaging, and delivery systems have enabled the expansion of SRS application in recent years, particularly for the treatment of patients with multiple brain metastases (MBM).^3^, ^4^ Single‐isocenter stereotactic radiosurgery has become an increasingly adopted approach for the treatment of multiple brain metastases (SI‐MBM SRS), offering substantial reductions in treatment time and resource utilization compared to multiple‐isocenter techniques.^5^, ^6^

However, these advantages come at the cost of increased geometric and dosimetric complexity, particularly for lesions located far from the isocenter. Previous studies have demonstrated that SI‐MBM SRS plans are more susceptible to rotational setup errors due to mechanical limitations, as well as due to the steep dose gradients and high levels of MLC modulation.^7^, ^8^, ^9^ Given the demand for high accuracy in SI‐MBM SRS, robust end‐to‐end (E2E) quality assurance (QA) is essential. E2E tests aim to evaluate the entire clinical workflow—from simulation and planning to dose delivery and verification—using phantoms and measurement systems that replicate clinical conditions. Among available dosimetry tools, 3D polymer gel dosimetry embedded within anatomically realistic phantoms provides a volumetric measurement of the delivered dose without requiring recalculation on a surrogate geometry. This has been shown to be particularly useful for complex SRS plans, as it enables direct comparison of measured and planned dose distributions.^10^, ^11^, ^12^

In parallel, plan complexity metrics (PCMs) have emerged as useful tools to quantify various aspects of treatment plan modulation, such as aperture irregularity, leaf motion variability, and the prevalence of small segments. These metrics have been linked to delivery accuracy and have been used to predict the outcome of patient‐specific QA in IMRT and VMAT.^13^, ^14^, ^15^ Recent studies have specifically investigated the role of complexity metrics in SI‐MBM SRS, suggesting that plan modulation can be associated with dosimetric robustness to delivery or setup errors.^16^, ^17^ However, the evaluation of how PCMs relate to 3D dosimetric accuracy in SI‐MBM SRS across platforms and geometries remains limited.

The aim of this multi‐institutional study is to investigate the relationship between target geometry and gamma passing rates (GPRs) derived from 3D end‐to‐end gel dosimetry in clinically realistic SI‐MBM SRS plans, as well as their correlation with established complexity metrics. By evaluating plans delivered across a variety of institutions, systems, and geometric configurations, this work aims to determine whether complexity and target geometry—based analysis can inform plan quality and delivery robustness in real‐world SRS practice.

## 2 MATERIALS AND METHODS

### 2.1 Study design

A total of 42 SI‐MBM SRS treatment plans were collected from different institutions and a total of 250 targets were studied. Each center performed end‐to‐end QA of a test case of their choice, using their standard procedures, equipment, and TPS configurations. As a result, the dataset included a broad spectrum of geometries, including different numbers of targets, variable target volumes, and diverse distances from isocenter. A summary of institutional demographics is provided in Table 1, while target‐specific features are shown in Table 2.

**TABLE 1:** Equipment of participating centers, including the numbers of the linear accelerators, the treatment planning systems and their combinations.

	*N* (42)	%
**Linac vendor**		
Varian (Novalis Tx, Truebeam STx, Edge, 2300IX)	29	69
Elekta (Synergy, Versa HD)	13	31
**Treatment planning system (TPS)**		
Varian (Eclipse)	10	24
Elekta (Monaco)	8	19
Brainlab (iPlan, Elements)	24	57
**Linac—TPS combination**		
Varian + Varian	10	24
Elekta + Elekta	8	19
Varian + Brainlab	19	45
Elekta + Brainlab	5	12

**TABLE 2:** SI‐MBM SRS plans characteristics included in the study across the different institutions.

Plans characteristics	Range
Number of targets	2‐16
Equivalent sphere diameter (mm)	2.3‐22.3
Distance from isocenter (mm)	0.05‐85.0

Abbreviations: SI‐MBM, single isocenter multiple brain metastases; SRS, stereotactic radiosurgery.

### 2.2 End‐to‐end workflow

**FIGURE 1:**
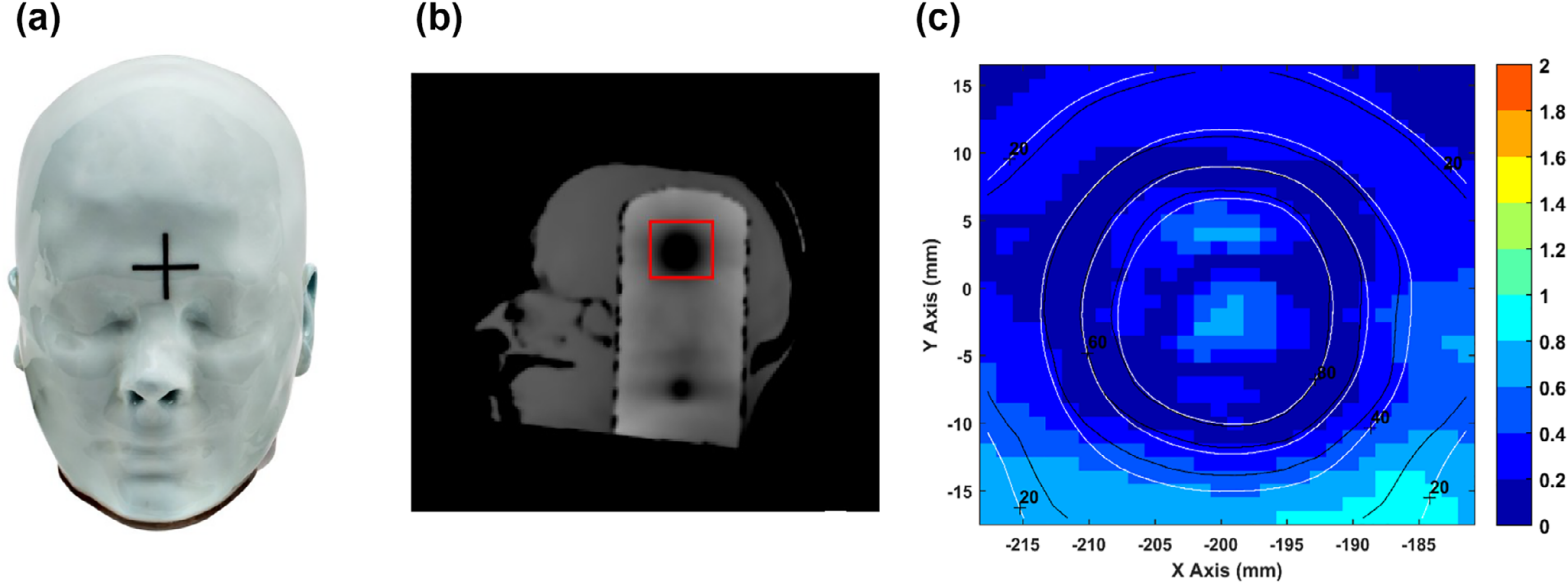
(a) 3D‐printed Prime™ head phantom (RTsafe P.C., Athens, Greece). (b) MR image of the phantom with the gel dosimeter insert and the region of interest. (c) 2D gamma index calculations: White isodose lines correspond to measured (gel) dose distributions, black isodose lines correspond to calculated (TPS) distributions.

To evaluate both spatial and dosimetric accuracy in a clinically relevant scenario, for the end‐to‐end tests the Prime™ head phantom (RTsafe P.C., Athens, Greece) was used, a 3D‐printed anthropomorphic phantom incorporating a polymer gel detector, as shown in Figure 1. This phantom's fabrication follows a patient‐specific design workflow, previously described by Makris et al.^18^ This approach involved reconstructing the external head contour and cranial skeletal anatomy directly from a previous patient's planning CT data. The resulting structure was manufactured using materials selected for their radiological equivalence to human bone under high‐energy photon beams. Prior studies have demonstrated the anatomical and dosimetric fidelity of this phantom model, supporting its application in stereotactic radiosurgery quality assurance^12^, ^19^, ^20^, ^21^ Treatment plans were generated based on structure sets that included the number and size of targets of the preference of each institution. Each plan was delivered according to each institution's routine clinical treatment workflows. These included the institution's immobilization, patient localization, image‐guidance, and treatment delivery procedures, replicating as closely as possible the clinical management of a patient. Thus, these strategies were not standardized across participating centers. The quantitative analyses presented in this study are based exclusively on the 3D gel dosimetry measurements performed with the Prime head phantom for all 42 treatment plans. Institution‐specific routine QA procedures were not collected or analyzed.

The gel formulation followed that of Saenz et al.,^12^ optimized for 3D dosimetry, presenting a linear relationship between the dose and the relaxation rate *R*_2_ in the range of 0‐12 Gy, as shown by Pappas et al.^22^ Thus, for single fraction SRS, the institutions used a prescription dose of 8 Gy, without exceeding the maximum dose of 12 Gy. In the cases of fractionated SRS, the irradiation of one of the total fractions was performed for the end‐to‐end test, to remain within the measurement range of the polymer gel. After irradiation, the phantom was kept at room temperature (∼20°C‐22°C) for 24 h to allow polymerization. MRI was performed using a 2D multi‐echo HASTE sequence with four echoes (starting at 36 ms, 400 ms intervals), high bandwidth (1220 Hz/pixel), and 20 averages to enhance SNR. *T*_2_ maps were generated and converted to *R*_2_ (1/*T*_2_) values, then rigidly registered to the planning CT for dose comparison. Image processing and dose reconstruction were done using validated MATLAB‐based routines.^10^, ^11^ Relative gel dosimetry was applied, enabling geometry offset calculations by directly comparing the gel‐derived and TPS‐calculated 3D dose distributions. Spatial offsets for each target were determined by comparing the center‐of‐mass (CoM) of the polymerized dose region with that of the planned high‐dose region. To account for dose gradients and image resolution, the CoM was averaged across multiple relative dose thresholds (50%‐70% of the prescription, in 5% steps), ensuring robust and target‐specific evaluation of spatial agreement. Global GPRs were calculated for each individual target with a 20% lower dose threshold for the acceptance criteria of 5%/1 mm, 5%/2 mm and 3%/2 mm.

### 2.3 Complexity metrics

To quantitatively assess the modulation of each plan, a set of 11 established PCMs regarding deliverability and accuracy was calculated. Deliverability metrics reflect the correlation of the delivery of a plan by a treatment machine with the mechanical and dosimetric variations inherent to the system, while accuracy metrics aim to capture complex MLC configurations that may undermine accurate delivery due to limitations in treatment planning system (TPS) modeling. Each metric was calculated based on the exported DICOM RT Plan files, utilizing UComX, a previously validated MatLab (MathWorks, Inc.)‐based free software package which supports plans generated from various treatment planning systems and delivery techniques.^23^

The deliverability metrics calculated included the Modulation Complexity Score (MCS) applied to VMAT,^13^ quantifying the MLC leaves complexity regarding the Aperture Area Variability (AAV)^14^ and the Leaf Sequence Variability (LSV).^14^ Additionally, Plan Irregularity (PI),^24^ showing the non‐circularity of the MLC aperture and the total Modulation Index (MIt),^25^ combining the speed and acceleration modulation of the MLC, the dose rate variation and gantry acceleration, were calculated.

The accuracy metrics included in this study were the Average Leaf Gap (ALG),^26^ showing the average distance between opposing leaf pairs, the Equivalent Field Size (EFS),^27^ representing the side of a square field equivalent to a rectangular one, the Tongue‐and‐groove index (TG),^28^ measuring the amount of leaf side lengths in the MLC apertures relative to the leaf pair openings, the Edge Metric (EM),^15^ meaning the amount of leaf side lengths in the MLC apertures to the total area, the Mean Asymmetry Distance (MAD),^29^ measuring the average distance between the centroid of the beam's eye view and the central axis of the MLC and the Small Aperture Score (SAS5mm),^29^ showing the fraction of leaf gaps smaller than 5 mm.

### 2.4 Data analysis and visualization

To assess the statistical differences in GPRs for all the acceptance criteria (5%/1 mm, 5%/2 mm and 3%/2 mm) and across subgroups, Mann‐Whitney *U* tests were used for non‐parametric pairwise comparisons. Specifically, these were applied across different TPS systems, linacs and their combination. Additionally, subgroup comparisons were performed based on interquartile (IQR) stratification for the number of targets per plan, targets’ equivalent diameter and their euclidean distance from the isocenter. Following the identification of statistically significant differences across IQR groups, ROC curve analysis was employed for all acceptance criteria to determine optimal classification thresholds for these variables, offering a more precise delineation of QA performance sensitivity. According to AAPM TG‐218,^30^ the 90% GPR was used as an action limit for the passing of the end‐to‐end QA test and QA failure was defined as the positive class. Youden J index was used to find the optimal threshold for the aforementioned parameters. This stratification enabled subgroup correlation analysis with Spearman's rank correlation coefficient (*r_s_*) between complexity metrics and the GPRs of SI‐MBM SRS plans with different geometric characteristics. Two‐sided *p*‐values were computed for each correlation, and Bonferroni correction was applied to adjust for multiple testing. The magnitude of the correlation was interpreted using standard cutoffs: values of | *r_s_* | < 0.20 were considered negligible, 0.20‐0.39 weak, 0.40‐0.59 moderate and ≥0.60 strong.

Statistical analysis and results visualization were conducted using the Python programming language (v. 3.9) leveraging code housed in the Pandas (v. 1.5.3) and Plotly (v. 5.3.0) libraries.

## 3 RESULTS

The calculated GPRs of Table 3, demonstrate that for the 5%/2 mm criteria 74.4% of the metastases were above the action limits of 90%. For the more stringent criteria of 5%/1 mm this proportion dropped to 43.6%, while for the 3%/2 mm criteria it fell to 65.6%, reflecting increased spatial deviations detection by the end‐to‐end test rather than dosimetric ones.

**TABLE 3:** GPRs calculated for the 250 targets through the end‐to‐end QA procedure for the three dose difference and distance to agreement acceptance criteria.

	GPRs		
Acceptance criteria	<50%	50%‐90%	≥90%
5%/1 mm	18	123	109
3%/2 mm	7	79	164
5%/2 mm	4	60	186

Abbreviation: GPRs, gamma passing rates.

### 3.1 Subgroup intercomparisons for different delivery and planning systems

Pairwise comparisons between the GPRs of treatment plans calculated by different TPS vendors, delivered by different linac models and all their combinations for the three acceptance criteria of 5%/2 mm, 5%/1 mm and 3%/2 mm were performed. The largest difference was observed when stratifying by TPS‐linac combinations, with Varian linacs utilizing the Brainlab TPS showing a 12% higher median GPR compared to Elekta linacs with the same TPS vendor, under the 5%/1 mm acceptance criteria. Nevertheless, none of the subgroup comparisons reached statistical significance (*p* > 0.05), partly due to the small subsample size. The maximum median differences from the pairwise comparisons and the distribution of GPR values across systems are provided in Table S1 and Figure S1.

### 3.2 Subgroup intercomparisons for different target geometries

Table 4 shows the maximum values of the median differences of the GPRs, along with their associated *p*‐values, of all the pairwise comparisons for plans stratified by target characteristics. Initially, GPRs were calculated on a per‐plan basis and grouped according to the number of targets. No statistically significant differences were observed for plans with fewer than three or more than seven targets (*p* > 0.05).

**TABLE 4:** The median differences of GPRs and their p‐values for three acceptance criteria between subgroups per target number, equivalent diameter of their volume and their euclidean distance from the isocenter. For each criterion the maximum observed median difference is reported of all the pairwise comparisons. Differences are expressed as Group1 minus Group2. The values with an asterisk represent statistically significant differences.

	Subgroup pair			
Acceptance criteria	Group1	Group2	Median GPR difference (%)	*p*‐value
**Target number**				
5%/2 mm	4‐6	7+	6.1	0.67
5%/1 mm	2‐3	7+	8.0	0.27
3%/2 mm	4‐6	7+	5.0	0.16
**Equivalent diameter (mm)**				
5%/2 mm	≤ 4.2	> 8.4	5.6*	<0.01
5%/1 mm	≤ 4.2	> 8.4	7.7*	0.014
3%/2 mm	≤ 4.2	> 8.4	9.1*	<0.01
**Distance from Iso range (mm)**				
5%/2 mm	0.05‐27.1	51.7‐85.0	5.6*	<0.01
5%/1 mm	0.05‐27.1	51.7‐85.0	14.2*	<0.01
3%/2 mm	0.05‐27.1	51.7‐85.0	6.0*	<0.01

Abbreviation: GPR, gamma passing rate.

Subsequently, GPRs were assessed on a per‐target basis, with plans stratified by interquartile ranges (IQRs) of two key geometric features, their distance from the isocenter and the diameter of the equivalent sphere of each target's volume. Stratification by IQR ensured balanced subgroup sizes for the assessment of whether these geometric parameters broadly influence GPRs. Statistically significant differences in end‐to‐end QA outcomes were found across all three gamma acceptance criteria for both classification variables.

**FIGURE 2:**
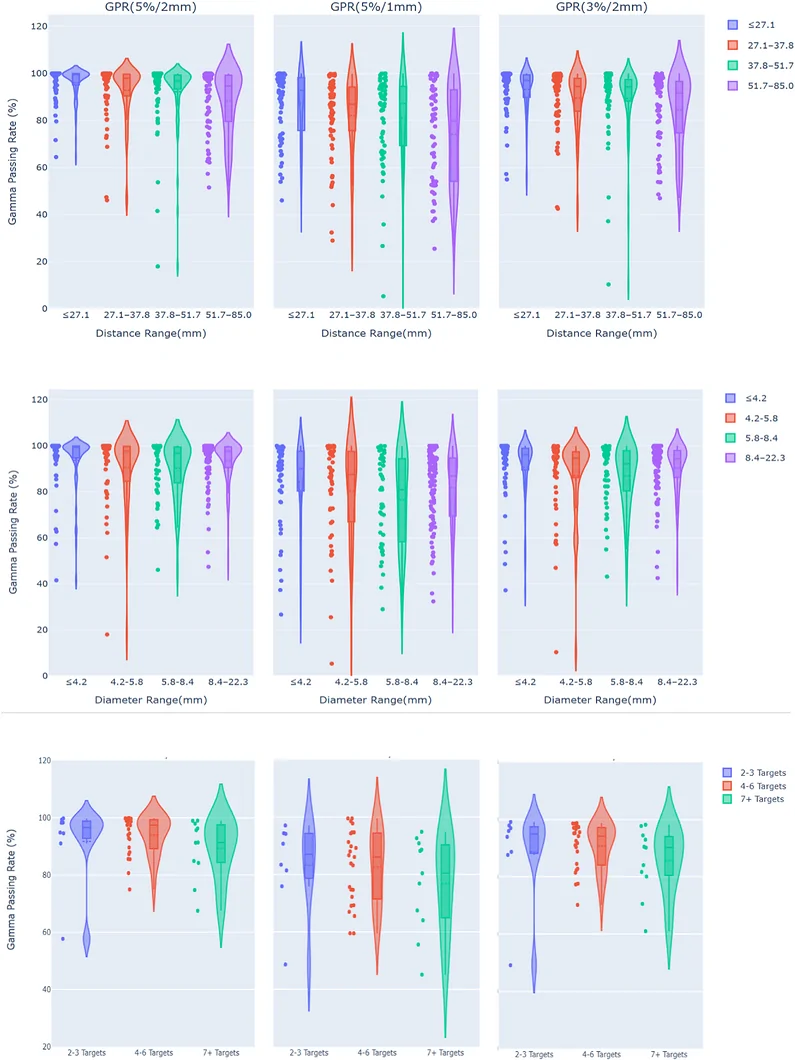
Interquartile ranges of GPRs per distance from isocenter, equivalent diameter and comparison of GPRs grouped by the number of targets for the 5%/2 mm, 5%/1 mm and 3%2 mm acceptance criteria. GPRs = Gamma Passing Rates.

The most pronounced differences were found between targets with a diameter of less than 4 mm and more than 8 mm (7.66%; 5%/1 mm), as well as for the ones located closer than 27 mm from the isocenter versus more than 52 mm further from it (14.15%; 5%/1 mm). The distributions of these subgroups are shown in the violin plots of Figure 2.

### 3.3 ROC analysis and comparisons with optimal thresholds

Since statistically significant differences were found between the subgroups stratified per equivalent diameter and distance to isocenter, ROC analysis was performed for these two parameters.

**FIGURE 3:**
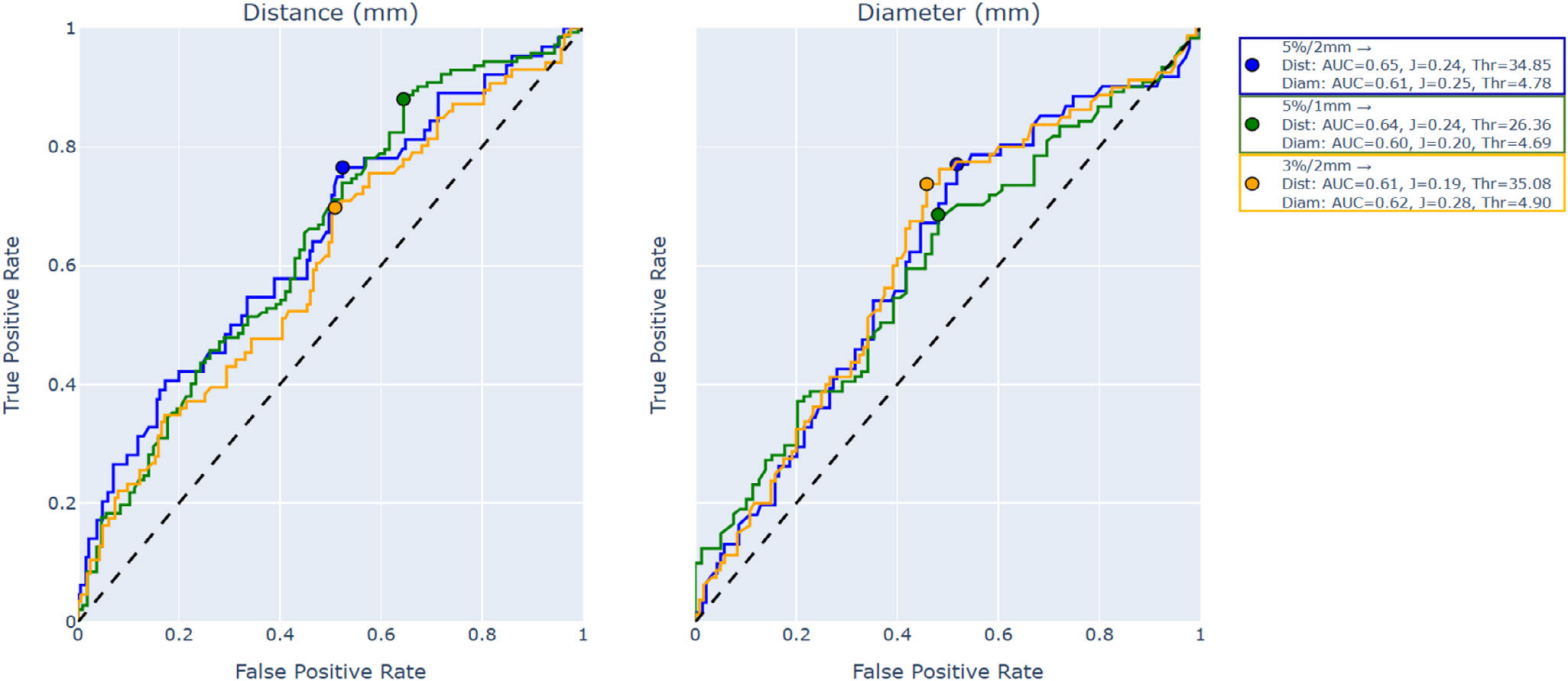
ROC curves of the distance from isocenter and the target volume equivalent sphere diameter for determining the thresholds above which the measurement would fail QA. The AUC values, thresholds and Youden J values for three gamma acceptance criteria are shown. ROC = Receiver Operating Characteristic; AUC = Area Under Curve; QA = Quality Assurance; GPR = Gamma Passing Rate.

The area under curve (AUC) of the equivalent diameter and the one of the distance from the isocenter had the highest values for the 5%/2 mm criterion, with the second one performing slightly better as a classifier (AUC: 0.60 and 0.65, respectively). The thresholds above which plans were more likely to fail QA were 4.78 mm for the equivalent diameter and 34.85 mm for the distance from isocenter. The ROC curves for all GPR acceptance criteria are shown in Figure 3.

**FIGURE 4:**
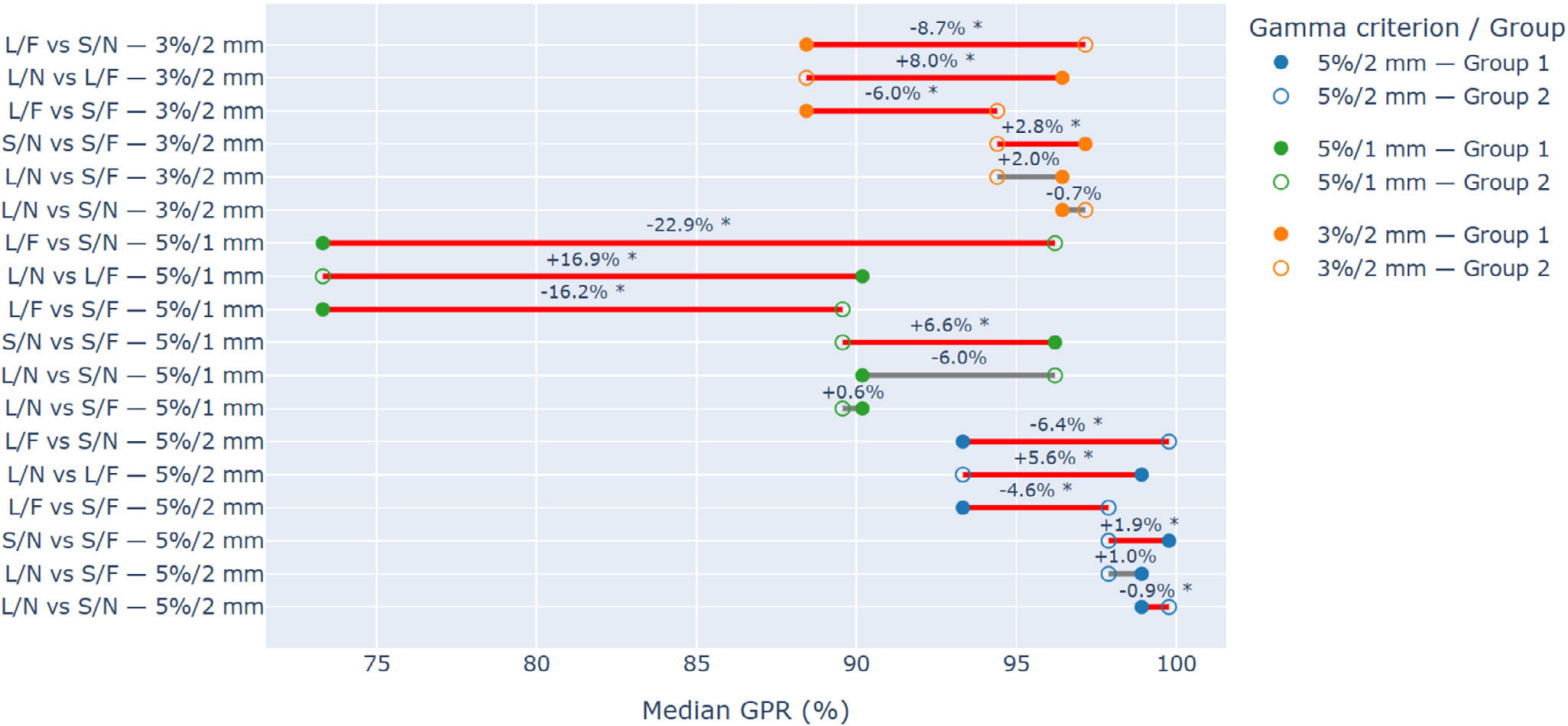
Dumbbell plots showing the differences between GPRs stratified into four groups based on the thresholds of 4.78 mm and 34.85 mm for the equivalent diameter and the distance from the isocenter of each target volume, accordingly. Each pairwise comparison was performed for all three acceptance criteria. Group 1 represents the left column, while Group 2 the right one. The red lines and the values with the asterisk (*) represent statistically significant median differences between the groups; the grey lines represent non statistically significant differences. GPRs = Gamma Passing Rates; L/F = Larger / Far; S/N = Smaller / Near; L/N = Larger / Near; S/F = Smaller / Far.

The thresholds were used to stratify the dataset in four categories, based on their equivalent diameter and their distance from isocenter, as Larger/Far (*N* = 67), Larger/Near (*N* = 52), Smaller/Far (*N* = 53), Smaller/Near (*N* = 28). The median differences were statistically significant (*p* < 0.01), with the greatest discrepancy observed between the Larger/Far and Smaller/Near groups. The median GPRs were greater for the Smaller/Near group by 6.45%, 22.89% and 8.72% for the acceptance criteria of 5%/2 mm, 5%/1 mm and 3%/2 mm, respectively. The dumbbell plots and the median differences between the subgroups are illustrated in Figure 4.

**FIGURE 5:**
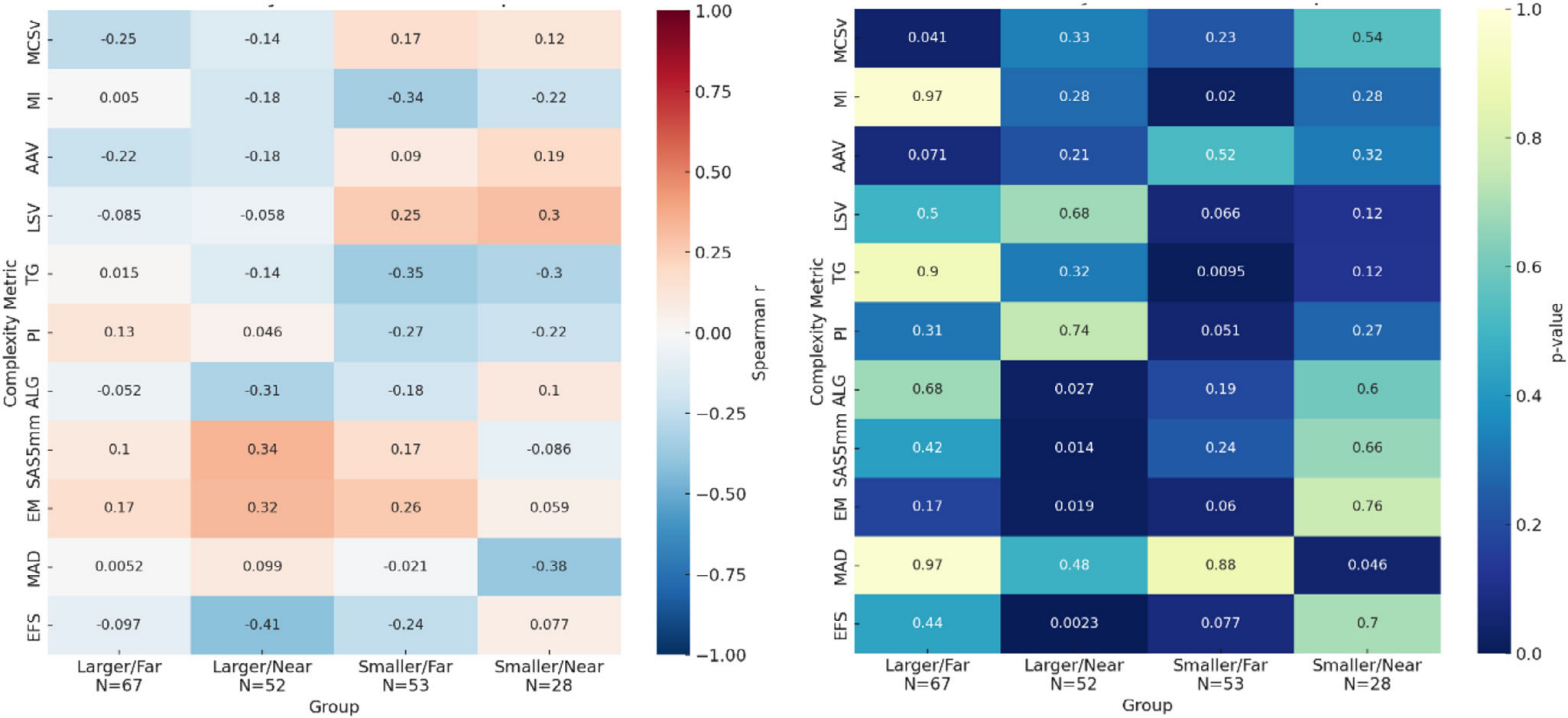
Spearman *r* correlation heatmap and *p*‐values heatmap of GPRs for the 5%/2 mm acceptance criteria and the complexity metrics, for the different subgroups of equivalent sphere diameter and distance to isocenter (Thresholds: 4.78 mm and 34.85 mm, accordingly). Correlation is significant at the 0.45% level (required for Bonferroni correction). GPR = Gamma Passing Rate; MCSv = Modulation Complexity Score for VMAT; MI = Modulation Index (total); AAV = Aperture Area Variability; LSV = Leaf Sequence Variability; TG = Tongue and Groove; PI = Plan Irregularity; ALG = Average Leaf Gap; SAS5mm = Small Aperture Score for leaf gaps < 5 mm; EM = Edge Metric; MAD = Mean Asymmetry Distance; EFS = Equivalent Field Size.

Figure 5 heatmaps show the relationships between GPRs for the 5%/2 mm criteria and the complexity metrics, along with their p‐values. The only parameter that showed a statistically significant relationship with GPRs (*p* < 0.0045) was EFS for the Larger/Near subgroup with a moderate correlation strength (*r* = −0.41, *p* = 0.0023). Nevertheless, the rest of the correlations were all classified as weak and non‐significant. This indicates the limited predictive value of plan complexity metrics for QA outcomes in a multi‐institutional setting characterized by geometric and procedural variability. Complexity metrics median and average values along with the standard deviation and their interquartile ranges are shown in Table 5.

**TABLE 5:** Complexity metrics median and average values for the SI‐MBM SRS plans, along with their interquartile ranges and the standard deviation.

Complexity metric	Median	Q1‐Q3	Average	SD
MCSv	0.35	[0.26‐0.47]	0.36	0.14
AAV	0.57	[0.39‐0.69]	0.54	36.85
LSV	0.72	[0.57‐0.77]	0.66	0.16
MI_t_	51.06	[37.15‐73.92]	62.49	0.14
TG	2.85	[2.44‐4.73]	3.79	2.10
PI	5.82	[4.79‐7.14]	7.03	4.81
ALG	10.00	[7.41‐12.45]	10.26	3.21
SAS5mm	0.16	[0.11‐0.27]	0.19	0.12
EM	0.19	[0.16‐0.24]	0.21	0.07
MAD	16.73	[13.75‐22.19]	17.68	6.01
EFS	16.27	[12.48‐18.24]	16.07	4.16

Abbreviation: SI‐MBM SRS, single isocenter multiple brain metastases stereotactic radiosurgery.

## 4 DISCUSSION

This study presents a comprehensive multi‐institutional assessment of SI‐MBM SRS using 3D gel‐based end‐to‐end QA, incorporating complexity analysis and geometric characterization across 42 plans and 250 targets. There was a significant variability in QA performance, with approximately a fourth of the plans failing to meet TG‐218^31^ action limit of 90% GPR (for the 5%/2 mm acceptance criteria). Prior studies have demonstrated that even high‐end technologies can be limited by isocentric constraints and rotational setup uncertainties when used for spatially dispersed lesions.^7^, ^9^ The diverse performance across institutions reinforces that end‐to‐end tests should be routinely performed for clinical practice evaluation, especially in geometrically challenging SRS contexts.

While system‐specific variations were observed, no statistically significant differences were found in gamma performance across linac models, TPSs, or their combinations. This is likely reflecting the combination of high intra‐group variability, relatively small subgroup sizes, and heterogeneity in local workflows. This aligns with the findings of Dimitriadis et al.,^31^ who reported no significant differences in dosimetric delivery accuracy among Gamma Knife, CyberKnife, and linacs during a national SRS audit for a single irregular shaped target. They emphasized that while system design and beam characteristics differ, institutional commissioning, planning approaches, and immobilization protocols are dominant drivers of variability, especially in linac‐based systems where practices vary widely. Eaton et al.^32^ demonstrated in their multi‐center planning intercomparison study for multiple brain metastases SRS, that although most platforms can achieve comparable conformity and gradient metrics for multiple brain metastases, greater variation was observed among linac‐based plans. This variation was attributed to differences in margin application, dose grid resolution, and planning strategies, rather than inherent platform limitations. This result is consistent with IROC audits showing that institutional practice and geometry outweigh vendor effects in determining end‐to‐end accuracy,^33^ as well as that differences in implementation, commissioning and modelling dominate in practical settings.

In contrast, target geometry emerged as a more consistent predictor of QA performance. Statistically significant differences in GPRs were found when stratifying targets by their distance from the isocenter and their equivalent diameter. Interquartile comparisons showed that targets further than 52 mm from the isocenter and those bigger than 8.4 mm had markedly lower GPRs, with differences up to 14% and 7.7%, accordingly, under 5%/1 mm criteria. The ROC analysis further supports the geometric dependency, with distance to isocenter (AUC up to 0.65) and equivalent diameter (AUC up to 0.60) performing as modest classifiers of QA failure. The derived thresholds (34.85 mm distance, 4.78 mm diameter) emerged as statistical indicators of geometric influence within this specific cohort. Rather than suggesting firm clinical boundaries, these thresholds are best viewed as exploratory tools for risk stratification and hypothesis generation in future work. The threshold of 3.5 cm for the targets’ distance from the isocenter aligns closely with the findings of previous studies, where spatial and dosimetric deviations exceeded acceptable limits for targets beyond 4 cm from the isocenter.^7^, ^12^

Counterintuitively, targets that the equivalent diameter of their volume was smaller than 5 mm tended to have higher median GPRs in comparison to bigger ones, as shown in Figure 2. Firstly, it is important to emphasize that all but two lesions had an equivalent diameter of less than 20 mm, the values of which were 21.33 mm and 22.34 mm, respectively. Thus, most of them fall within the smallest size category defined in the RTOG protocol 90‐05^34^ and can be susceptible to small fields related uncertainties. The statistically significant differences between the subgroups could be due to the fact that plans involving larger targets may have required more complex field shapes and higher modulation in order to meet healthy brain tissue or organs at risk constraints, potentially resulting in less robust plans. Inaccuracies in beam modeling or mechanical performance can be more pronounced in highly modulated plans, explaining the lower GPRs in such cases. These findings can also suggest that, in multi‐institutional studies, the parameter of equivalent diameter may be more susceptible to inter‐planner variability, as larger or higher number of targets often necessitate steeper dose gradients and more intricate modulation strategies. The differences likely reflect a combined effect of target diameter, geometric offset from the isocenter, and total irradiated volume during multi‐target optimization. Therefore, the role of size in this context should be interpreted cautiously and viewed primarily as a surrogate marker for underlying geometric and planning complexity, rather than as an independent predictor of QA performance.

In order to assess the combined effect of the two geometric parameters on the QA outcomes, we proceeded with their comparison into a four‐tiered classification. As shown in Figure 4, the “Larger/Far” targets had the lowest GPRs, while “Smaller/Near” targets had the highest, with the difference in GPR between these two classes exceeding 22% in the 5%/1 mm criterion. When analyzed independently, both size and distance contributed substantially to QA variability. Increasing distance for large targets (L/N vs. L/F) and increasing size at large distance (S/F vs. L/F) each reduced GPRs by approximately 16‐17% under the 5%/1 mm criterion. These results indicate that size and distance exert comparable effects when considered separately, but their interaction is additive, yielding the poorest QA outcomes when both adverse conditions coincide. Our results suggest that even in a setting of vendor‐diverse, with highly varying clinically delivered plans, target geometry remains a significant factor in QA performance variability in SI‐MBM SRS.

The influence of geometric parameters on QA outcomes in linac‐based SRS has been investigated from multiple perspectives. Through assessment of target coverage loss in dose‐volume terms, previous studies^35^, ^36^, ^37^ have demonstrated that in single‐isocenter, multi‐target SRS, smaller, off‐axis lesions are particularly susceptible to coverage loss in the presence of rotational errors, reflecting their steep dose gradients and limited margins. By contrast, Price et al.^38^ observed that in a range of 2‐5.6 cm of targets’ equivalent diameter, larger targets exhibited lower passing rates in measurement‐based QA of VMAT SBRT cases, with approximately 26% of plans failing institutional action limits; although the correlation between PTV volume and GPR was weak (*r* = −0.31), they attributed this counterintuitive trend to the more complex dose distributions of larger targets, proximity to organs‐at‐risk, and resolution limitations of the chamber array employed. Our results are in line with the latter observation: we identified reduced gamma GPR for large, off‐axis targets, while near‐isocenter targets showed less size dependence. However, our use of high‐resolution gel dosimetry eliminates the detector sampling limitations cited by Price et al.,^38^ suggesting that the degradation we observed is attributed to a higher degree of machine delivery uncertainties and planning, or beam modeling challenges. Furthermore, our multi‐institutional dataset encompassed vendor‐diverse TPS and linac platforms with heterogeneous structure sets and planning strategies, which likely introduced additional variability compared to single‐institution studies or clinical audits. Further investigation of the effect of distance‐to‐isocenter and target size in QA measurements in a more standardized set, will reduce heterogeneity of target geometries across institutions.

The four subgroups were further analyzed regarding their correlation with complexity metrics. Previous studies^13^, ^39^ have reported strong associations between certain metrics and gamma agreement in controlled settings, however, our results suggest that, under real‐world, multi‐institution conditions, complexity alone is not a dominant determinant of QA performance. Our evaluation of eleven plan complexity metrics across 42 multi‐institutional SI‐MBM SRS plans did not reveal strong or consistent correlations with GPRs under any of the tested criteria. Only Equivalent Field Size (EFS) showed a significant, moderate correlation (*r* = −0.41, *p* = 0.0023) within one subgroup (Larger/Near). This finding is in line with a prior large‐scale phantom audit study of Glenn et al.,^33^ who assessed 16 different fluence‐ and aperture‐based metrics in more than 300 head‐and‐neck IMRT/VMAT phantom irradiations, reported universally weak or absent correlations with measured dosimetric performance. Similarly, several earlier investigations^14^, ^24^ concluded that modulation complexity scores and related indices do not reliably predict IMRT QA outcomes, despite their clear physical relevance as descriptors of leaf motion, aperture variability, and modulation strength. In SRS, this disconnect may be even more pronounced. Standard complexity metrics, derived from IMRT and VMAT paradigms, are blind to off‐isocenter beam angles, non‐coplanar arcs or small field dosimetry—all of which are central to SI‐MBM delivery. Additionally, the negligible explanatory power for QΑ outcomes of complexity metrics such as EM or EFS calls into question the utility of applying them to SRS plans evaluation, since their values will be affected by the small irregular segments required in such plans, due to the small irradiated volume and the need for high gradients. Consequently, there is a need to refine or adapt complexity metrics for SRS workflows, potentially by integrating them with geometry‐based descriptors. Despite these limitations, reducing plan complexity is important for limiting delivery uncertainties and increasing robustness. A recent multi‐institutional study from May et al.^16^ on the robustness of MBM SRS plans to setup errors reported that lower complexity correlated significantly with increased robustness, showing that the D99% of up to 20% fewer targets failed the specified threshold for less complex plans. Thus, in regards of plan quality, complexity metrics should not only be interpreted as predictors of QA outcomes, but also as indicators of plan sensitivity to errors. The multi‐institutional nature and heterogeneity of the dataset, along with the use of 3D gel‐based dosimetry in anatomically accurate phantoms are distinguishing features of this study and key to its clinical relevance. Unlike 2D QA systems, gel dosimeters provide high spatial resolution dose readout and capture spatial dose deviations across the entire lesion volume, while also allowing for direct comparison with TPS calculations without needing dose recalculation or interpolation. Additionally, the evaluation of test cases that were planned according to each institutions clinical workflows, allows to capture the interplay between plan geometry, system performance, and delivery accuracy under conditions that more closely mirror routine practice in comparison to standardized datasets. However, the same strengths introduce challenges.

Several limitations of this study should be considered when interpreting the findings. First, the multi‐institutional design intentionally incorporated substantial variability in treatment planning systems, linac platforms, commissioning practices, planning strategies, image‐guidance workflows, and patient positioning protocols to reflect real‐world clinical practice. Consequently, the observed differences in end‐to‐end QA performance represent the cumulative effect of the entire treatment chain rather than the influence of any single factor. In particular, although distance from the isocenter emerged as a significant predictor of reduced GPRs, the present end‐to‐end methodology cannot distinguish between the contributions of geometric uncertainties, image‐guidance and patient positioning accuracy, TPS beam‐modeling limitations, or machine delivery characteristics. Likewise, detailed information regarding immobilization techniques, image‐guidance protocols and the application of six‐degree‐of‐freedom couch corrections was not collected in a standardized manner across all participating institutions, precluding subgroup analyses based on localization strategy. Furthermore, the broad heterogeneity in target number, lesion size, target location, and institutional planning approaches, while representative of current clinical practice, inevitably reduced the statistical power of some subgroup comparisons and may have obscured more subtle associations between plan complexity metrics and QA outcomes. This heterogeneity may explain the variability in results and the lack of system‐specific significance, but it also highlights the urgent need for harmonized QA strategies in SI‐MBM SRS. A recent study from Thomann et al.^40^ on the evaluation of 23 radiotherapy centers different approaches in SI‐MBM SRS in Germany, Austria and Switzerland, reported high protocol variability not only regarding the different treatment delivery systems, but also parameters such as calculation grid size, minimum accepted target diameter or margin strategies. This target heterogeneity was evident in our study, too. As more institutions adopt this technique, shared guidelines that incorporate geometry‐based risk stratification will be necessary to guarantee reliable and effective clinical use.

## 5 CONCLUSIONS

This multi‐institutional end‐to‐end study using high‐resolution 3D gel dosimetry provides novel evidence on the relative contributions of plan complexity and target geometry to QA performance in single‐isocenter, multi‐metastases SRS. While no strong or consistent correlations were observed between established complexity metrics and GPRs, target size and distance to isocenter were found to be significant determinants of dosimetric agreement, especially for off‐axis lesions. These results indicate that geometric considerations may outweigh conventional modulation‐based indices in predicting QA outcomes under clinically realistic, vendor‐diverse conditions, underscoring the need to refine existing complexity metrics or integrate them with geometry‐ and delivery‐specific parameters tailored for SRS. Future work in more standardized datasets will be essential to separate geometry, complexity, and machine or beam modeling effects, and to guide the development of harmonized QA strategies that ensure safe and reliable clinical adoption of SI‐MBM SRS.

## AUTHOR CONTRIBUTIONS

All authors helped in the development of the manuscript, editing, drafting of the paper, the design of the methodology, measurements, analysis, review of data, and approved the final version to be published.

## CONFLICT OF INTEREST STATEMENT

The authors declare no conflicts of interest.

## Supporting information



Supporting Information: acm270758‐sup‐0001‐TableS1.docx

Supporting Information: acm270758‐sup‐0002‐FigureS1.pdf

Supporting Information: acm270758‐sup‐0003‐SuppMat.docx

## Data Availability

The data that support the findings of this study are available from the corresponding author upon reasonable request.
