# Supplementary material for: Assessing the role of plan complexity and target geometry through multi‐institutional gel based end‐to‐end QA in multi‐focal single isocenter stereotactic radiosurgery
Source: J Appl Clin Med Phys. 2026 Sep 24;27(10):e70758. doi: 10.1002/acm2.70758 (PMC13601935; doi:10.1002/acm2.70758)
Supplement: Supplementary file 1 [file ACM2-27-e70758-s001.pdf]

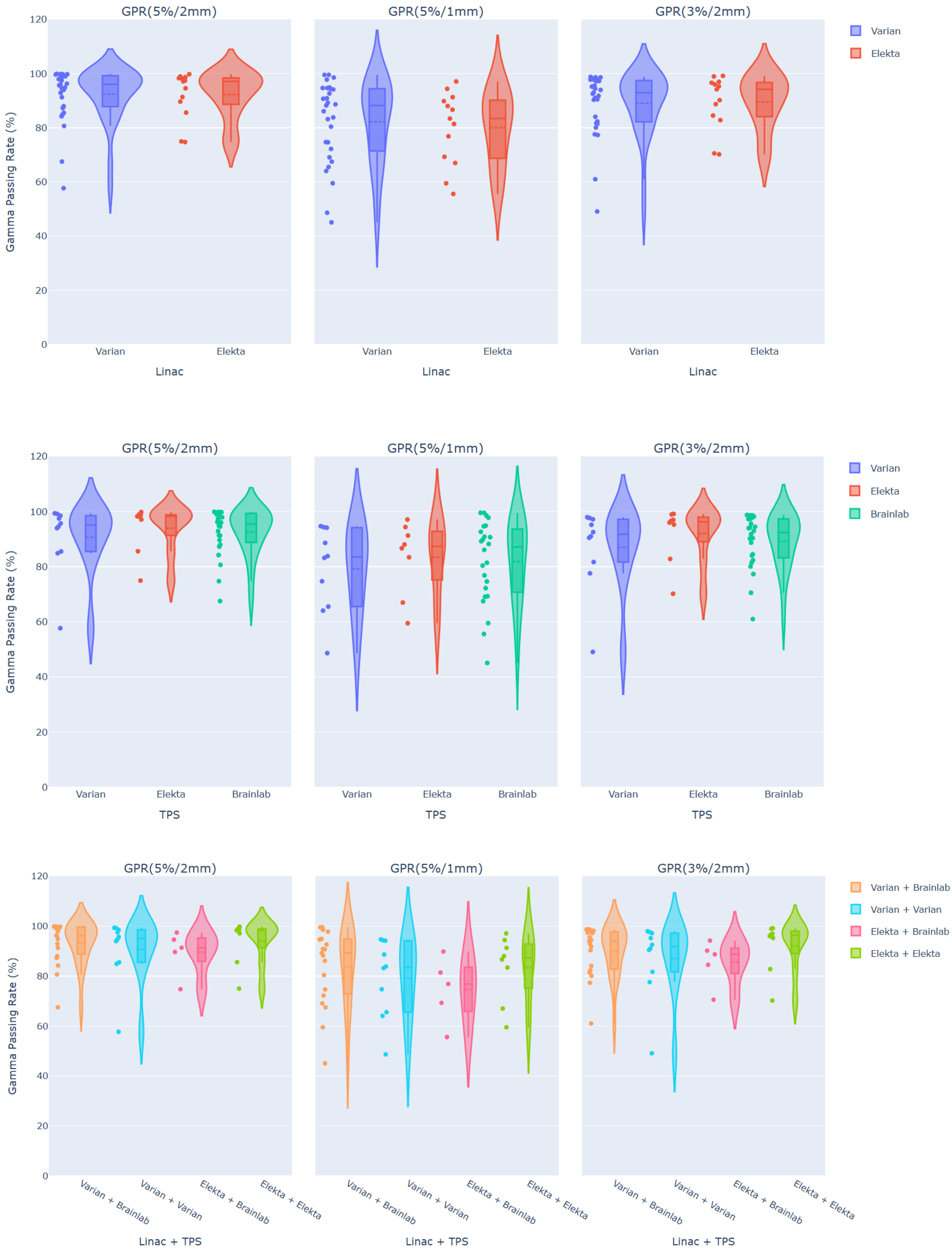
GPR(5%/2mm)
GPR(5%/1mm)
GPR(3%/2mm)
Gamma Passing Rate (%)
0
20
40
60
80
100
120
Varian
Elekta
Linac
Varian
Elekta
Brainlab
TPS
Varian + Brainlab
Varian + Varian
Elekta + Brainlab
Elekta + Elekta
Linac + TPS
