# Supplementary material for: Assessing the role of plan complexity and target geometry through multi‐institutional gel based end‐to‐end QA in multi‐focal single isocenter stereotactic radiosurgery
Source: J Appl Clin Med Phys. 2026 Sep 24;27(10):e70758. doi: 10.1002/acm2.70758 (PMC13601935; doi:10.1002/acm2.70758)
Supplement: Supplementary file 2 [file ACM2-27-e70758-s002.docx]

**Table S1:** The median differences of GPRs and their p-values for three acceptance criteria between subgroups of different linac models, TPS vendors and their combinations. For each criterion the maximum observed median difference is reported of the pairwise comparisons. Differences are expressed as Group1 minus Group2.

| Acceptance Criteria | | Subgroup Pair | | | | Median GPR Difference (%) | | p-value |
| --- | --- | --- | --- | --- | --- | --- | --- | --- |
|  | Group 1 | | Group 2 | |  | |  | |
|  | | Linac | | | |  | |  |
| 5%/2mm | | Varian | | Elekta | | -1.1 | | 0.67 |
| 5%/1mm | | Brainlab | | Elekta | | 4.8 | | 0.45 |
| 3%/2mm | | Varian | | Elekta | | -1.2 | | 1.0 |
|  | | TPS | | | |  | |  |
| 5%/2mm | | Brainlab | | Elekta | | -2.9 | | 0.66 |
|  |  | Varian | | Elekta | | -3.2 | | 0.41 |
| 5%/1mm | | Brainlab | | Varian | | 3.6 | | 0.58 |
|  |  | Varian | | Elekta | | -3.8 | | 0.57 |
| 3%/2mm | | Brainlab | | Elekta | | -3.9 | | 0.27 |
|  |  | Varian | | Elekta | | -4.5 | | 0.32 |
|  | | Linac + TPS | | | |  | |  |
| 5%/2mm | | Elekta/Brainlab | | Elekta/Elekta | | -6.9 | | 0.13 |
| 5%/1mm | | Varian/Brainlab | | Elekta/Brainlab | | 12.4 | | 0.18 |
| 3%/2mm | | Elekta/Brainlab | | Elekta/Elekta | | -7.6 | | 0.14 |

Abbreviations: GPR = Gamma Passing Rate; TPS = Treatment Planning System
