# Supplementary material for: Assessing the role of plan complexity and target geometry through multi‐institutional gel based end‐to‐end QA in multi‐focal single isocenter stereotactic radiosurgery
Source: J Appl Clin Med Phys. 2026 Sep 24;27(10):e70758. doi: 10.1002/acm2.70758 (PMC13601935; doi:10.1002/acm2.70758)
Supplement: Supplementary file 3 [file ACM2-27-e70758-s003.docx]

**Figure S1:** GPRs violin plots stratified per linac, TPS and their combinations for the 5%/2mm, 5%/1mm and 3%/2mm acceptance criteria. The solid line presents the median value, while the dotted line the mean value of their distributions.
Abbreviations: GPRs = Gamma Passing Rates; TPS = Treatment Planning System
